# Mind–Body Exercises for PTSD Symptoms, Depression, and Anxiety in Patients With PTSD: A Systematic Review and Meta-Analysis

**DOI:** 10.3389/fpsyg.2021.738211

**Published:** 2022-01-18

**Authors:** Lin Zhu, Long Li, Xiao-zhi Li, Lin Wang

**Affiliations:** ^1^School of Wushu and Art, Nanjing Sport Institute, Nanjing, China; ^2^Department of Physical Education, Southeast University, Nanjing, China; ^3^Department of Physical Education, Wuhan University of Technology, Wuhan, China

**Keywords:** mind-body exercise, depression, anxiety, exercise prescription, PTSD, HPA axis

## Abstract

**Objectives:**

This study aims to systematically analyze the effects of mind–body exercises on post-traumatic stress disorder (PTSD) symptoms, depression, and anxiety in patients with PTSD. Furthermore, it intends to provide scientific evidence-based exercise prescriptions.

**Methods:**

Chinese (i.e., China National Knowledge Infrastructure, VIP Database for Chinese Technical Periodicals, and Wanfang) and English (i.e., Web of Science, PubMed, the Cochrane Library, and EMBASE) databases were used as data sources to search for studies on the effects of mind–body exercises on symptoms associated with patients with PTSD from January 1980 to November 2020. After a rigorous screening, 16 eligible randomized controlled trials (RCTs) were included in the meta-analysis.

**Results:**

Mind–body exercises exerted a significant effect on PTSD symptoms [standard mean difference (SMD) = −0.41, 95% confidence interval (CI) −0.64 to −0.19, *p* < 0.001], depression (SMD = −0.35, 95% CI: −0.55 to −0.15, *p* < 0.001), and anxiety (SMD = −0.31, 95% CI: −0.74 to −0.12, *p* < 0.001) among patients with PTSD. Subgroup analysis demonstrated that 60–150 min per session for 8–16 weeks of mindfulness was more effective in improving symptoms in patients with PTSD under 45 years of age compared with other subgroups. For depression, 150–180 min of yoga exercises once per week was effective. For anxiety, the frequency, timing, duration, and type of mind–body exercises that are most effective in relieving anxiety in patients with PTSD cannot be determined at this time due to the limited number of eligible RCTs.

**Conclusions:**

Mind–body exercises were found to be significantly effective in improving PTSD symptoms, depression, and anxiety in patients with PTSD. Therefore, they can be used as an adjunct to intervention for symptoms of patients with PTSD. However, this conclusion requires further confirmation through additional scientific and objective RCTs.

**Systematic Review Registration:**

Unique Identifier: INPLASY2020120072.

## Introduction

The sudden outbreak of the coronavirus disease (COVID-19) pandemic in 2019 triggered a global mental health crisis. Internationally, the incidence of COVID-19 was related to increased depression, anxiety, excessive stress, drug abuse, and increased suicidal ideation among the general population (Czeisler et al., 2020). Moreover, the COVID-19 outbreak exerted a serious impact on life, work, and studies, which led to several negative psychological reactions (Shi et al., 2020). Specifically, the high contagiousness of COVID-19 caused widespread negative emotions, such as anxiety, helplessness, and panic (Bo et al., 2020; El-Hage et al., 2020). One of the typically negative psychological outcomes of the epidemic was post-traumatic stress disorder (PTSD) (Fekih-Romdhane et al., 2020; Liu et al., 2020). For example, the author in (Xiong et al., 2020) analyzed the literature on PTSD in the context of the *new crown* epidemic and found that the incidence of PTSD ranged from 7 to 53.8% (Xiong et al., 2020). Therefore, exploring the effect of mind–body exercises on the relief of PTSD symptoms despite the continued global spread of COVID-19 can not only provide effective exercise prescriptions for patients with PTSD but also alleviate anxiety and panic as a result of the epidemic. Moreover, it can even prevent the occurrence of PTSD during the COVID-19 epidemic.

Events that lead to PTSD symptoms can also occur indirectly, such as when a person hears about the details or scenes of the death or serious injury of a loved one or a friend (Forbes et al., 2007). PTSD is typically caused by extensive traumatic experiences and is, therefore, common among survivors of war or major natural disasters, people recovering from major epidemics, victims of violence, and veterans. Surveys demonstrated that the prevalence of PTSD in the general population ranges from ~5 to 10% (Forbes et al., 2007). A census in the United States illustrated that more than half of the population (60.7% of men and 51.2% of women) reported experiencing traumatic events (i.e., flood, fire, natural disaster, life-threatening accidents, rape or sexual assault, witnessing a death, or severe injury). Among these people, 7.8% suffer from PTSD throughout their lives, whereas women are twice as likely to suffer from PTSD symptoms than men (Kessler et al., 2005).

Studies have demonstrated that the effects of PTSD are multifaceted. According to the Diagnostic and Statistical Manual of Mental Disorders (DSM-5), a diagnosis of PTSD is composed of four major symptom clusters, namely, negative cognition and emotion; reminders to avoid events; excessive arousal; and intense revisiting of traumatic events through destructive memories and nightmares (Hegberg et al., 2019). In addition to the characteristic symptoms of PTSD, changes in brain structure that lead to functional changes (Hayes et al., 2012a; Miller et al., 2017) and cognitive impairment (Hayes et al., 2012b; Woon et al., 2017) are also well-documented. People with PTSD may suffer from severe issues with social survival and interpersonal communication due to the characteristics of the diseases, such as long-term invasiveness, avoidance, and excessive arousal, which results in impaired quality of life (Schnurr et al., 2006). In addition, patients with PTSD tend to exhibit activities in the higher sympathetic nerve and lower parasympathetic nerve (Fonkoue et al., 2018). Compared with healthy people, patients with PTSP display symptoms of low heart rate variability (Streeter et al., 2012). Although psychological interventions and traditional medication were proven effective in treating symptoms associated with PTSD (Cukor et al., 2009; Lancaster et al., 2016), many problems emerged regarding the side effects and therapeutic effects of drugs. Moreover, patients with PTSD typically report several obstacles, such as treatment cost, motivation, stigma, and access to care (Sayer et al., 2009; Dickstein et al., 2010). Although exercises present hindrances and drawbacks, such as limited motivation, self-efficacy, or exercise time, they are widely available, low-cost, and low-risk forms of intervention that can effectively prevent the side effects associated with traditional pharmacological and psychological intervention methods.

In recent years, adjuvant treatments for patients with PTSD and other trauma-related diseases have increasingly received scholarly attention. In exploring methods for promoting physical and mental health, many studies have shifted their focus on mind–body exercises. They have mainly utilize physical, mental, psychological, and behavioral exercises, such as breathing adjustment, physical exercise, meditation (Wynn, 2015; Zhang et al., 2019b; Zou et al., 2019a). This form has emphasized the holy trinity of exercise, i.e., combining the body, mind, and breathing. At the same time, it has the advantage of improving physical and mental health (Chan et al., 2013; Cushing and Braun, 2018; Zou et al., 2018a,b; Zhang et al., 2019a). Recently, various modalities of integrative mind–body intervention, which are increasingly employed in the treatment of PTSD, have emerged. Moreover, an increasing body of evidence has illustrated that mind–body interventions have exerted a positive impact on the psychological status, physical health, stress reduction, and quality of life of individuals with PTSD (Gordon et al., 2004, 2008; Grodin et al., 2008; Descilo et al., 2010; Bormann et al., 2011; Staples et al., 2011; Kearney et al., 2012; Hegberg et al., 2019). In 2010, 39% of people with PTSD reported using complementary and alternative medicine interventions, which includes various mind–body exercises that combine breathing and stretching exercises, such as tai chi, yoga, qigong, and meditation (Sapolsky et al., 2000). In fact, previous studies have demonstrated that mind–body exercises may positively influence PTSD-related symptoms through psychological and neurophysiological mechanisms, such as exposure and desensitization to internal arousal cues, improved cognitive function, normalization of the hypothalamic–pituitary–adrenal (HPA) axis function, motor-induced neuroplasticity, and reduction of inflammatory markers (McEwen, 2002; Yehuda and Golier, 2009; Streeter et al., 2012). In addition, emerging evidence supports the notion that mind–body exercises can effectively improve the symptoms of stress-related diseases through neurological and biological mechanisms (Bernardi et al., 2001; Vaiva et al., 2004; Brown and Gerbarg, 2009). Therefore, mind–body exercises may improve symptoms associated with PTSD, such as depression and anxiety, and may have the potential for use as an adjunctive treatment for patients with PTSD.

This study aims to review the effects of mind–body exercises (i.e., yoga, tai chi, qigong, and meditation) on PTSD symptoms. These four intervention methods require more active participation but less environment and related facilities. Moreover, they exhibit a high degree of *portability* and a huge dissemination potential for easy promotion and implementation (Zhou et al., 2019). Recently, numerous studies evaluated the specific effects of mind–body exercises on the presence of mood disorders related to PTSD symptoms, depression, and anxiety in patients with PTSD. However, these studies reported varying impacts on PTSD symptoms due to variability in sample selection (i.e., content, age, and gender) and the implementation of specific interventions (i.e., timing, frequency, method, and duration). Therefore, the current meta-analysis intends to explore the intrinsic regulatory mechanisms of mind–body exercises in patients with PTSD and to assess their effects on the symptoms and mood disorders associated with patients with PTSD to provide appropriate exercise prescriptions. Meanwhile, the exercise intervention program proposed by this study is expected to alleviate the mental health burden associated with COVID-19 at the global level.

## Materials and Methods

### Search Strategy

The current study is registered on the INPLASY website under registration number INPLASY2020120072 (https://inplasy.com/inplasy-2020-12-0072/). The literature was obtained from English (i.e., Web of Science, EMBASE, Cochrane Library, and PubMed) and Chinese (i.e., China National Knowledge Infrastructure, Wanfang, and VIP Database for Chinese Technical Periodicals) databases. A search was conducted to identify randomized controlled trials (RCTs) published between January 1, 1980 and November 30, 2020 in any language on the effects of mind–body exercises on patients with PTSD. The search terms include “mindfulness” OR “mind–body exercise” OR “yoga” OR “stretching” OR “tai chi” OR “qigong” OR “meditation.” PTSD-related terms include “PTSD,” “post-traumatic stress disorder,” “trauma” AND “depression,” “anxiety disorder,” “mood disorder,” “depressive disorder,” AND “anxiety disorder.”

### Inclusion Criteria

The criteria for the inclusion of eligible articles were as follows. (1) Studies should be categorized as RCTs. (2) The experimental sample should consist of adults (18 years or older) with a psychiatrist-confirmed DSM-Fourth Edition-Text Revision diagnosis of PTSD. The results of their physical examination should indicate that they are eligible for participation in exercise programs along with consent to participate. (3) The sample should consist of experimental groups involving mind–body exercises (e.g., mindfulness, yoga, tai chi, qigong, and meditation) vs. control groups with different interventions (e.g., treatment as usual, no physical activity, and maintenance of daily life). (4) The primary outcome measures should include relevant data on PTSD symptoms, depression, and anxiety. Lastly, (5) the language of the literature should be only Chinese or English.

### Exclusion Criteria

Studies were further screened according to the following exclusion criteria: (1) repeated studies; (2) Abstract articles only (no full text available), review studies, and non-RCT studies; (3) studies with vague or no data available for analysis; (4) studies with participants with substance dependence, psychiatric disorders, or prescriptions for alpha- or beta-blocking drugs; and (5) studies on massage, acupuncture, and other similar passive mind–body interventions.

### Collection of Studies

Two researchers (Lin Zhu and Long Li) first read and screened the titles and abstracts of the documents and eliminated those that did not meet the inclusion criteria. If the studies met the inclusion criteria, then their full text was further evaluated and screened. In the case of disagreement between the two researchers during the screening process, a third researcher (Lin Wang) was invited to discuss with them to further verify whether the articles under disagreement met the inclusion criteria.

### Data Extraction

Details of the literature that met the inclusion criteria were extracted, which include the first author of the study, year of publication, information about the participants in the experiment group (i.e., sample size, mean age/age range, and dropout rate), the specific design of the experiment (i.e., frequency of mind–body exercises, duration of each session, intervention period, and follow-up), outcome measures and analysis of relevant indicators, and the final study results. Meanwhile, quantitative data were also extracted from the literature, such as sample size, mean and standard deviation for measurement indicators related to PTSD, depression, and anxiety in terms of mind–body exercises and control groups.

### Methodological Quality Assessment

Two investigators independently assessed the quality of studies that met the inclusion criteria using the modified Physical Therapy Evidence Database scale (Zou et al., 2019b). The modified assessment criteria consisted of nine items (i.e., randomization of sample selection; hidden allocation; similarity at baseline; assessor blinding; ≤ 15% dropout rate; intention-to-treat analysis; between-group comparison of intervention and control groups; point and variability measures; and independent exercise intervention) with high final scores indicating high-quality methods used in the study.

### Study Analysis Method

This study used Stata 14.0 for meta-analysis. First, an overall forest map was drafted; heterogeneity was analyzed; and further regression and subgroup analyses were conducted. According to the Cochrane Handbook for Systematic Reviews of Interventions, we selected fixed- or random-effect meta-analysis according to the potential effect of the intervention on the outcome. We calculated the SMD and 95% confidence intervals (95% CI), where SMDs were considered small, medium, and large differences at intervals of 0.2–0.49, 0.5–0.79, and 0.8, respectively. Moreover, *I*^2^ values represent low (25%), medium (50%), and high (75%) heterogeneity (Higgins et al., 2003). The random-effect model was used for meta-analysis when the heterogeneity test *I*^2^ ≥ 50%, whereas the fixed-effect model was used when *I*^2^ <50%. Moreover, regression analysis was conducted to investigate the degree of experimental heterogeneity. Categorical variables (e.g., age of the sample, type of intervention, frequency of exercises, periodicity, and duration of each) were subject to subgroup analysis with the objective of determining which subgroup was more effective in improving symptoms associated with patients with PTSD and to provide a theoretical basis for later experiments.

## Results

### Selection of Studies

**Figure 1:**
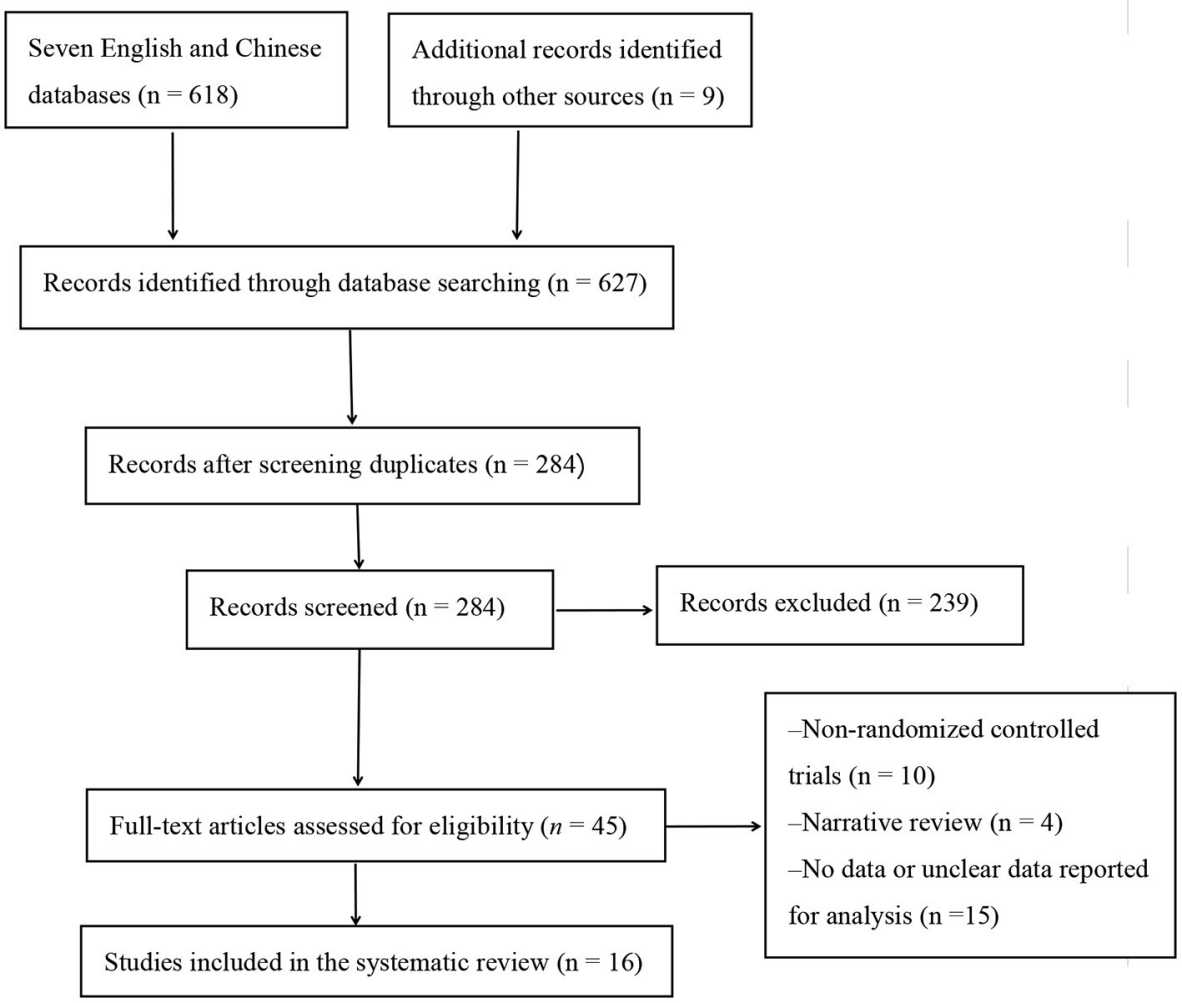
Flow chart of study selection.

The initial literature was identified from seven English and Chinese databases (*n* = 618) and other resources (*n* = 9; Figure 1). After removing duplicate studies and those that did not meet the inclusion criteria, 284 articles remained. After a further screening, 45 papers were retained. A careful reading of the full text of the remaining 45 papers led to the exclusion of 29 studies, which included non-RCTs (*n* = 10), review articles (*n* = 4), and no or unclear measurements (*n* = 15). Finally, after a final rigorous screening, 16 eligible studies were included in the meta-analysis.

### Characteristics of Eligible Studies

Table 1 presents the 16 eligible RCTs (Carter et al., 2013; Kearney et al., 2013; Kim et al., 2013; Omidi et al., 2013; Dick et al., 2014; Mitchell et al., 2014; Seppälä et al., 2014; van der Kolk et al., 2014; Jindani et al., 2015; Polusny et al., 2015; Quiñones et al., 2015; Kelly and Garland, 2016; Possemato et al., 2016; Goldsteina et al., 2017; Reinhardt et al., 2017; Huberty et al., 2020). In total, the selected studies included 871 participants, where the smallest and largest samples were 21 (Seppälä et al., 2014) and 116 (Polusny et al., 2015), respectively. The age of the experimental sample ranged from 18 to 65 years. The shortest and longest intervention periods were 1 and 16 weeks, respectively (Quiñones et al., 2015). The experimental group included various interventions, such as mindfulness and yoga. The control group of the experiment maintained regular daily life, usual treatment, and toning exercises (Table 1).

**Table 1:** Summary of the characteristics of the included studies.

**References**	**Country**	**Sample size (attrition rate)**	**Gender (male)**	**Mean age or age range**	**Duration (W)**	**Experimental group intervention**	**Control group intervention**	**Outcome assessments**	**Follow-up**
Mitchell et al. (2014)	United States	38 (0%)	0	18–65 (44.37)	12	1 × 75 min/week; hatha yoga	Regular daily life	PCL/STAI/CES-D	Yes
Jindani et al. (2015)	Canada	80 (37.5%)	9	18–64 (41)	8	1 × 90 min/week; Kundalini yoga (KY)	Regular daily life	PCL/DASS 21/ISI/PSS	No
Seppälä et al. (2014)	United States	21 (4.7%)	21	T = 28.09 ± 2.91 C = 29.20 ± 6.66	1	7 × 18 min/week Sudarshan Kriya yoga	Regular daily life	PCL/MASQ/GDD/GDA	Yes
Quiñones et al. (2015)	Colombia	100 (9%)	65	No	16	2 × 60 min /week Satyananda Yoga	Mandatory ordinary assistance protocol designed by ACR	PCL	No
Reinhardt et al. (2017)	United States	38 (52.6%)	34	T = 44.12 ± 13.97 C = 46.58 ± 12.66	10	2 × 90 min/week; yoga sessions	No treatment	PCL/CAPS	Yes
Huberty et al. (2020)	United States	60 (50%)	0	>18	12	1 × 150 min/week; yoga	60 min/week of toning exercises	STAI/PTSD/PHQ-9/PSQI	Yes
Goldsteina et al. (2017)	United States	47 (0%)	38	T = 47.42 ± 15.94 C = 46.31 ± 14.37	12	3 × 60 min/week exercise sessions (aerobics)exercise, yoga, mindfulness)	Regular daily life	CAPS	Yes
van der Kolk et al. (2014)	United States	64 (6.25%)	0	T = 41.5 ± 12.2 C = 44.3 ± 11.9	10	1 × 60 min/week; yoga intervention	TAU	CAPS/BDI-II	No
Dick et al. (2014)	United States	38 (31.6%)	26	44.37 ± 12.37	12	1 × 75 min/week; yoga intervention	Regular daily life	PCL	Yes
Kim et al. (2013)	United States	22 (14.3%)	0	T = 47.6 ± 7.7 C = 45.0 ± 10.0	8	8 × 60 min/week; MBX sessions	Regular daily life	PCL	Yes
Omidi et al. (2013)	IR Iran	62 (0%)	37	39–59	8	7 × 120 min/week; MBSR	TAU	BRUMS	No
Carter et al. (2013)	Canada	31 (19.5%)	25	T = 58.5 ± 3.8 C = 58.4 ± 4.8	1	22 h of yoga over 5 days; SKY intervention	No intervention reported	CAPS/CES-D/PCL-M	Yes
Possemato et al. (2016)	United States	62 (29.0%)	54	T = 46.3 ± 16.4 C = 47.4 ± 16.2	4	1 × 90 min/week BMT	TAU	PCL-S/PHQ-9/CAPS	Yes
Kearney et al. (2013)	United States	47 (6.3%)	37	T = 52 ± 13.4 C = 52 ± 11.7	8	1 × 150 min/week MBSR + TAU	TAU	PCL-S/PHQ-9/BADS	Yes
Polusny et al. (2015)	United States	116 (14.6%)	98	T = 57.6 ± 10.4 C = 59.4 ± 9.2	8	1 × 150 min/week MBSR	Present-centered group therapy	PCL/PHQ-9/BADS	Yes
Kelly and Garland (2016)	United States	45 (13.3%)	39	41.5 ± 14.6	8	1 × 150 min/week MBSR	Wait-list control	PCL-C/BDI-II/RSQ	Yes

*W, week; T, trait group; C, control group; TAU, treatment as usual; PCL, PTSD Checklist; STAI, State Trait Anxiety Inventory; CES-D, Center for Epidemiologic Studies-Depression Scale; ISI, Insomnia Severity Index; DASS 21, Depression, Anxiety, and Stress Scale; PSS, Perceived Stress Scale; MASQ, Mood and Anxiety Symptoms Questionnaire; GDA, General Distress-Anxiety; GDD, General Distress-Depressive; DASS-42, Depression Anxiety and Stress Scale; PSQI, Pittsburgh Sleep Quality Index; CAPS, Clinician Administered PTSD Scale; PHQ-9, Patient Health Questionnaire-9; HAM-D/-A, Hamilton Depression/Anxiety Rating scales; BDI-II, Beck Depression Inventory-II; SCL-90-R, symptom checklist-90-revision; BMT, brief mindfulness training; MBSR, mindfulness-based stress reduction; RSQ, Relationship Structures Questionnaire; MBX, mindfulness-based stretching and deep breathing exercises*.

### Methodological Quality Assessment

The methodological quality scores for the selected studies ranged from 5 to 8 (Table 2). All studies were eligible for the following: RCTs with similar baseline characteristics and studies with between-group comparisons; point and variable descriptive measures; and independent mind–body exercise interventions. Only three studies implemented concealed allocation and assessor blinding (Carter et al., 2013; Quiñones et al., 2015; Possemato et al., 2016). The dropout rates in all six studies were higher than 15% (Carter et al., 2013; Dick et al., 2014; Jindani et al., 2015; Possemato et al., 2016; Reinhardt et al., 2017; Huberty et al., 2020), whereas five studies did not conduct isolated exercise interventions (Kim et al., 2013; Mitchell et al., 2014; Seppälä et al., 2014; van der Kolk et al., 2014; Goldsteina et al., 2017).

**Table 2:** Study quality assessment for eligible randomized controlled trials.

**References**	**Item 1**	**Item 2**	**Item 3**	**Item 4**	**Item 5**	**Item 6**	**Item 7**	**Item 8**	**Item 9**	**Score**
Mitchell et al. (2014)	1	0	1	0	1	1	1	1	0	6
Jindani et al. (2015)	1	0	1	0	0	0	1	1	1	5
Seppälä et al. (2014)	1	1	1	1	1	1	1	1	0	8
Quiñones et al. (2015)	1	0	1	0	1	1	1	1	1	7
Reinhardt et al. (2017)	1	0	1	0	0	0	1	1	1	5
Huberty et al. (2020)	1	0	1	0	0	1	1	1	1	6
Goldsteina et al. (2017)	1	0	1	0	1	1	1	1	0	6
van der Kolk et al. (2014)	1	0	1	0	1	1	1	1	0	6
Dick et al. (2014)	1	0	1	0	0	0	1	1	1	5
Kim et al. (2013)	1	0	1	0	1	1	1	1	0	6
Omidi et al. (2013)	1	0	1	0	1	1	1	1	1	7
Carter et al. (2013)	1	1	1	1	0	1	1	1	1	8
Possemato et al. (2016)	1	1	1	1	0	0	1	1	1	7
Kearney et al. (2013)	1	0	1	0	1	1	1	1	1	7
Polusny et al. (2015)	1	0	1	0	1	1	1	1	1	7
Kelly and Garland (2016)	1	0	1	0	1	0	1	1	1	6

*Item 1 = randomization; Item 2 = concealed allocation; Item 3 = similar baseline; Item 4 = blinding of assessors; Item 5 = ≤ 15% dropouts; Item 6 = intention-to-treat analysis; Item 7 = between-group comparison; Item 8 = point measure and measures of variability; Item 9 = isolate exercise intervention; 1 = explicitly described and present in details; 0 = absent, inadequately described, or unclear*.

### Meta-Analysis of Outcome Indicators

#### Effects of Mind–Body Exercises on Patients With PTSD

**Figure 2:**
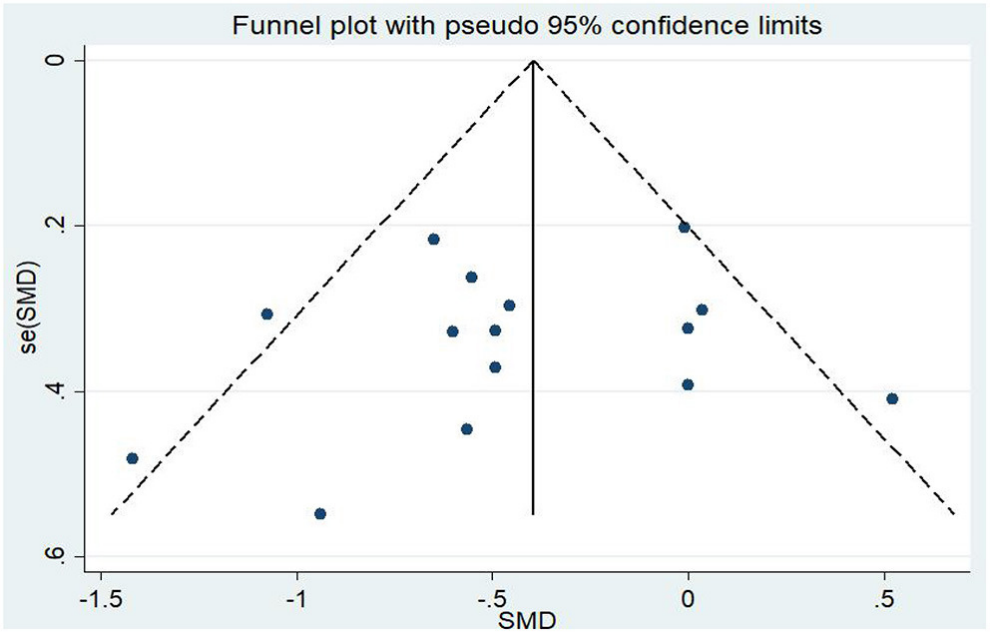
Funnel plot of publication bias for PTSD.

**Figure 3:**
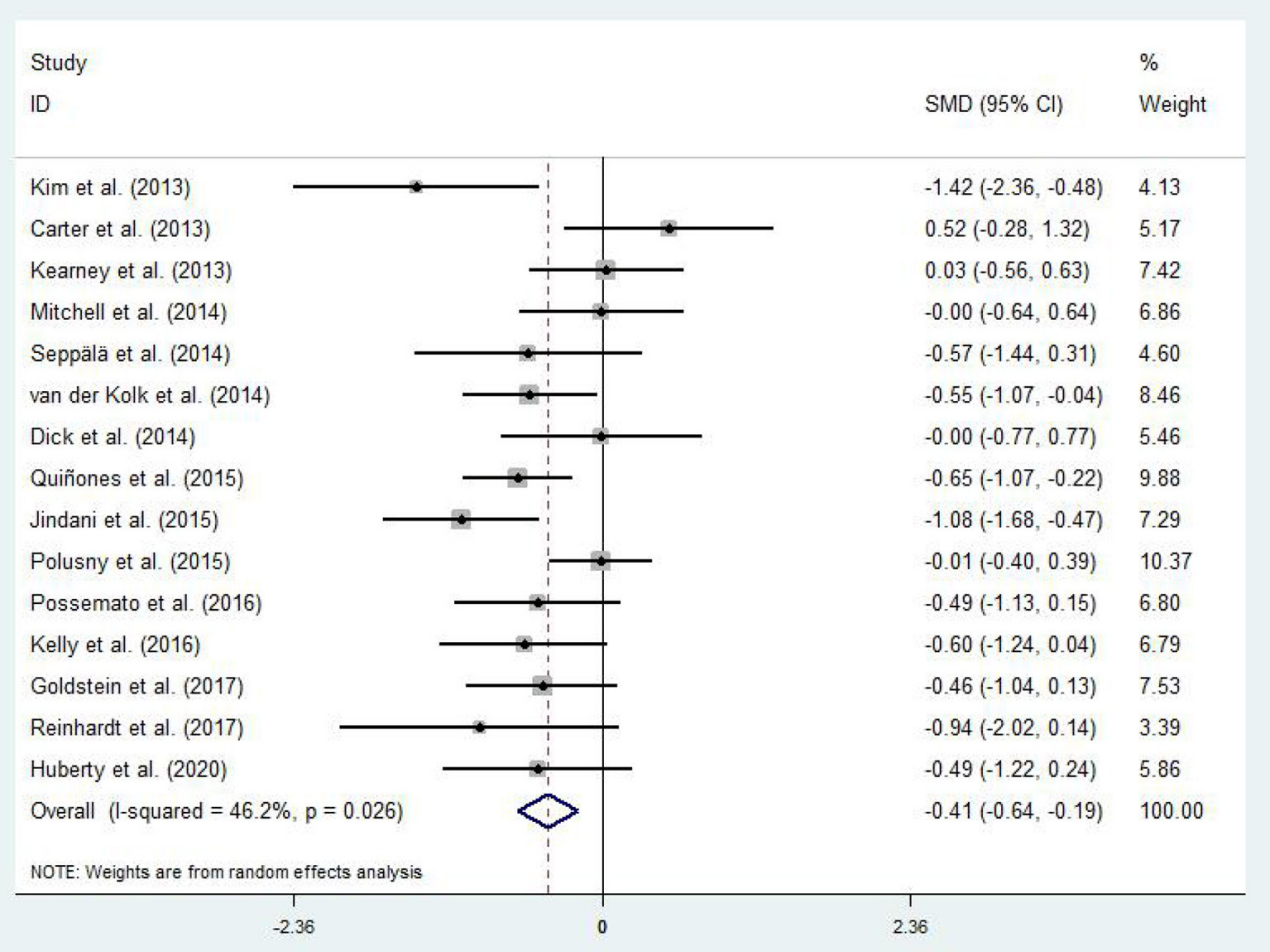
Effect of mind–body exercises on PTSD.

Differences in PTSD symptoms in 15 (Carter et al., 2013; Kearney et al., 2013; Kim et al., 2013; Dick et al., 2014; Mitchell et al., 2014; Seppälä et al., 2014; van der Kolk et al., 2014; Jindani et al., 2015; Polusny et al., 2015; Quiñones et al., 2015; Kelly and Garland, 2016; Possemato et al., 2016; Goldsteina et al., 2017; Reinhardt et al., 2017; Huberty et al., 2020) of the 16 articles that met the criteria were compared before and after the experimental and control groups. The asymmetric funnel plot indicates the presence of three outliers (Figure 2). The study employed the random-effect model due to the moderate heterogeneity in the literature (*p* = 0.026, *I*^2^ = 46.2%; Figure 3). The results of the meta-analysis of the 15 studies revealed that mind–body exercises significantly improved PTSD symptoms (SMD = −0.41, 95% CI: −0.64 to −0.19, *p* < 0.001).

Sensitivity analysis of heterogeneity and elimination revealed that the studies by Jindani et al. (2015), Reinhardt et al. (2017), and Kim et al. (2013) presented considerable bias. Thus, we excluded them to perform a meta-analysis of the remaining RCTs. The results demonstrated that heterogeneity was reduced (*I*^2^ = 17.5%, *p* = 0.282; SMD = −0.28, 95% CI: −0.46, −0.10) with significant differences between the groups (*p* < 0.001).

Covariates, such as age, gender, frequency, time, duration, and event are likely to be influencing factors for PTSD symptoms in patients with PTSD. Table 3 presents the results of the regression of covariates for patients with PTSD. Specifically, no significant effects were observed for age (95% CI: −0.0325892, 0.0913166, *p* = 0.29), gender (95% CI: −0.0185921, 0.0153174, *p* = 0.821), frequency (95% CI: −0.1941011, 0.3065732, *p* = 0.602), time (95% CI: −0.0053595, 0.8061784, *p* = 0.906), duration (95% CI: −0.0885154, 0.230738, *p* = 0.318), or event (95% CI: −0.8915781, 2.12068, *p* = 0.954).

**Table 3:** Covariate regression analysis of PTSD symptoms in patients with PTSD.

**_ES**	**Coef**.	**Std. err**.	***t***	***p* > *t***	**(95% CI)**	
Age	0.0293637	0.0253188	1.16	0.29	−0.0325892	0.0913166
Gender	−0.0016374	0.006929	−0.24	0.821	−0.0185921	0.0153174
Frequency (times/week)	0.056236	0.1023074	0.55	0.602	−0.1941011	0.3065732
Time (min)	0.006322	0.004774	1.32	0.234	−0.0053595	0.0180035
Duration (week)	0.0711113	0.065236	1.09	0.318	−0.0885154	0.230738
Event	−0.0426999	0.3469182	−0.12	0.906	−0.8915781	0.8061784
_cons	−3.026597	1.167725	−2.59	0.041	−5.883918	−0.1692755

In this study, the factors influencing the experimental results were categorized into different groups according to category, such as age, gender, frequency, time, duration, and type of exercise (Table 4). The results of the subgroup analysis were as follows: (1) age: mind–body exercises were beneficial for patients with PTSD who were aged less 45 years and with PTSD symptoms. (2) Gender: when males outnumber females, mind–body exercises exerted a more significant effect on PTSD symptoms. (3) Frequency: mind–body exercises conducted 1–3 times per week was more effective in improving PTSD symptoms than 4–8 times per week. (4) Time: mind–body exercises for a duration of 60–150 min was effective in improving PTSD symptoms. (5) Duration: in the subgroup for exercise intervention duration, the improvement of symptoms in patients with PTSD was more significant with an 8–16-week intervention. (6) Event: the effect of mindfulness exerted significant effects on PTSD symptoms.

**Table 4:** Subgroup analysis of PTSD symptoms.

**Group**	**Subgroup**	**N**	**SMD**	**95%CI**		***p***	***I* ^2^**
Age	<45	6	−0.491	−0.821	−0.16	0.178	34.50%
	≥45	7	−0.31	−0.699	0.078	0.026	58.10%
Gender	Male > female	8	−0.291	−0.578	−0.005	0.094	42.60%
	Male < female	7	−0.566	−0.911	−0.222	0.088	45.60%
Frequency (times/week)	1 time/week	8	−0.315	−0.603	−0.027	0.084	44.20%
	2–3 times/week	5	−0.662	−0.927	−0.397	0.496	0.00%
	4–8 times/week	2	−0.008	−1.073	1.057	0.073	68.90%
Time (min)	60 ≤ min ≤ 90	9	−0.578	−0.837	−0.320	0.162	32.00%
	90 < min ≤ 150	4	−0.19	−0.494	0.114	0.312	15.80%
	150 < min ≤ 180	2	−0.008	−1.073	1.057	0.073	68.90%
Duration (week)	1–8 weeks	3	−0.189	−0.858	0.48	0.103	56.00%
	8 weeks	5	−0.545	−1.074	−0.015	0.004	74.20%
	8–16 weeks	7	−0.458	−0.686	−0.229	0.544	0.00%
Event	Mindfulness	8	−0.365	−0.64	−0.09	0.114	39.80%
	Yoga	7	−0.464	−0.852	−0.075	0.04	54.50%

#### Effects of Mind–Body Exercises on Patients With PTSD and Depression

**Figure 4:**
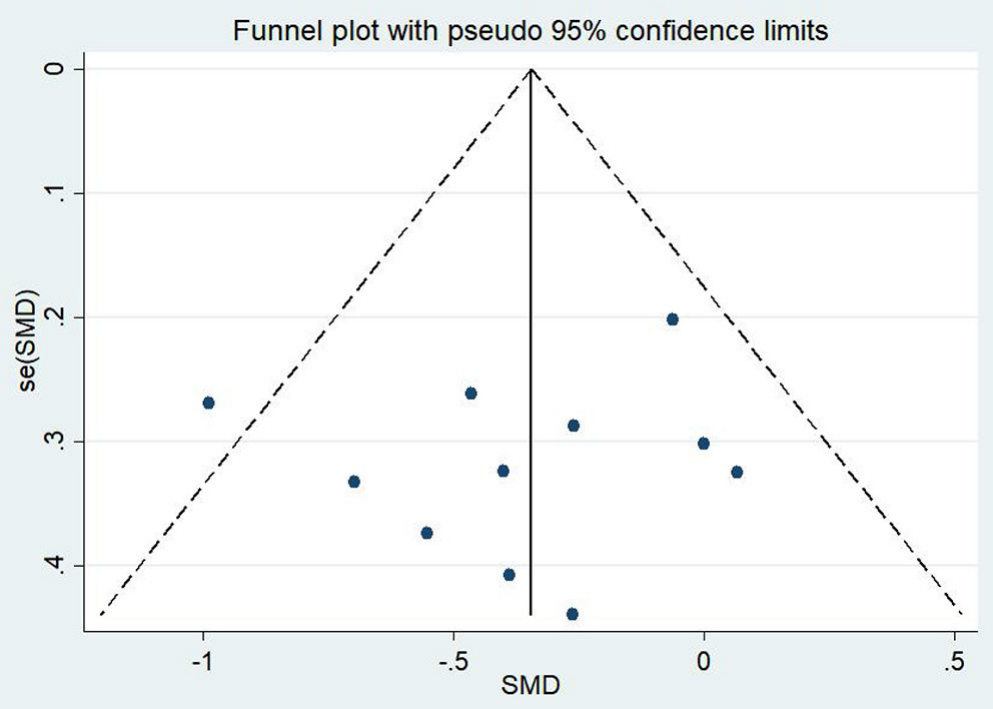
Funnel plot of publication bias for depression.

**Figure 5:**
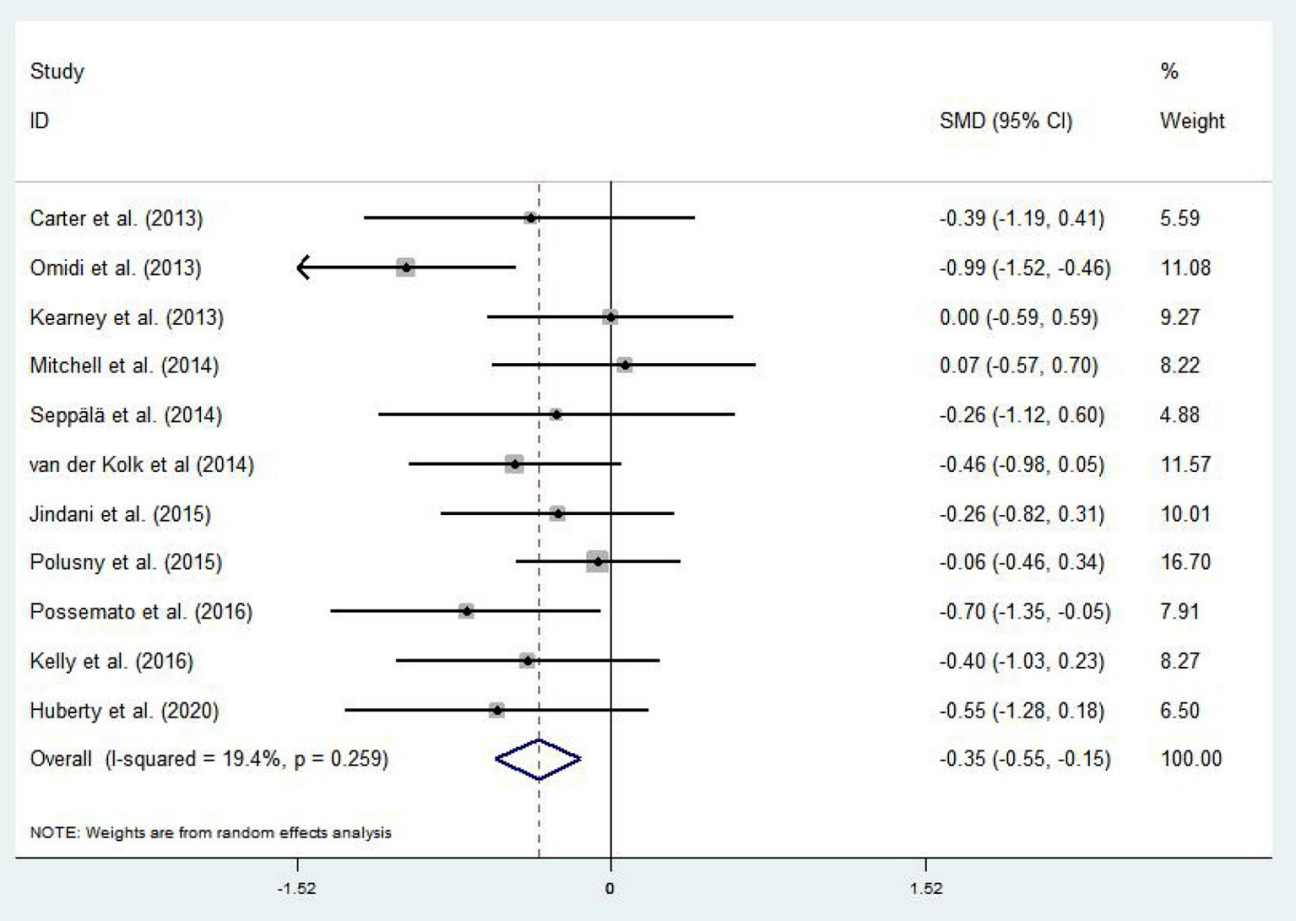
Effect of mind–body exercises on depression.

A total of 11 articles (Carter et al., 2013; Kearney et al., 2013; Omidi et al., 2013; Mitchell et al., 2014; Seppälä et al., 2014; van der Kolk et al., 2014; Jindani et al., 2015; Polusny et al., 2015; Kelly and Garland, 2016; Possemato et al., 2016; Huberty et al., 2020) evaluated the effects of mind–body exercises on depression in patients with PTSD. Figure 4 illustrates an asymmetric funnel plot and indicates the presence of one outlier. The test of heterogeneity of the included literature depicted a low level of heterogeneity (*p* = 0.259, *I*^2^ = 19.4%). Therefore, the meta-analysis was performed using the fixed-effect model (Figure 5). The results demonstrated that mind–body exercises significantly improved depression in patients with PTSD (SMD = −0.35, 95% CI: −0.55 to −0.15, *p* < 0.001).

Sensitivity analysis of heterogeneity and elimination revealed that the studies by Omidi et al. (Omidi et al., 2013) exhibited considerable bias; thus, it was excluded. Afterward, analysis was continued for the remaining RCTs. The results indicated reduced heterogeneity (*I*^2^ = 3.2%, *p* = 0.643, SMD = −0.26, 95% CI: −0.45, −0.07), whereas the difference between the experimental and control groups remained significant (*p* < 0.001).

Covariates including the participants' basic profile (e.g., age and gender) and the intervention method (i.e., frequency, time, duration, and type of exercise) may be factors that influence the degree of depression in patients with PTSD. Table 5 provides the results of the covariate regression for depressive symptoms in patients with PTSD. For depression, no significant effects were observed for age (95% CI: −0.0699061, 0.0646416, *p* = 0.909), gender (95% CI: −0.0165347, 0.0190792, *p* = 0.835), frequency (95% CI: −0.2637994, 0.0550195, *p* = 0.129), time (95% CI: −0.0072837, 0.0172842, *p* = 0.286), duration (95% CI: −0.1327683, 0.2251, *p* = 0.472), or event (95% CI: −0.4794182, 1.172184, *p* = 0.274).

**Table 5:** Covariate regression analysis of depression in patients with PTSD.

**_ES**	**Coef**.	**Std. err**.	***t***	***p > t***	**(95% Cl)**	
Age	−0.0026322	0.021139	−0.12	0.909	−0.0699061	0.0646416
Gender	0.0012722	0.0055954	0.23	0.835	−0.0165347	0.0190792
Frequency(times/week)	−0.1043899	0.0500902	−2.08	0.129	−0.2637994	0.0550195
Time (min)	0.0050003	0.0038599	1.3	0.286	−0.0072837	0.0172842
Duration (week)	0.0461659	0.0562254	0.82	0.472	−0.1327683	0.2251
Event	0.3463829	0.2594862	1.33	0.274	−0.4794182	1.172184
_cons	−1.438363	0.8840877	−1.63	0.202	−4.251925	1.375198

In all eligible studies, we performed a subgroup analysis based on the underlying profile of the participants and the intervention method (Table 6). The results are as follows. (1) Age: mind–body exercises were beneficial for the improvement of depressive symptoms in patients with PTSD aged more than 45 years. (2) Gender: when females outnumbered the males, the improvement in depressive symptoms in patients with PTSD is more significant. (3) Frequency: the effect of performing mind–body exercises once per week or 4–8 times per week was more effective for depression symptoms in patients with PTSD. (4) Time: Exercises for 60–90 min or 150–180 min per session were beneficial for depressive conditions in patients with PTSD. (5) Duration: An intervention period of 1–8 or 8–16 weeks exerted a significant effect on depressive symptoms in patients with PTSD. (6) Event: For patients with PTSD and depression, the effect of yoga exerted significant effects.

**Table 6:** Sub-group analysis of depression in patients with PTSD.

**Group**	**Subgroup**	**N**	**SMD**	**95%CI**		***p***	***I^2^***
Age	<45	6	−0.433	−0.677	−0.189	0.209	30.10%
	≥45	4	−0.2	−0.476	0.076	0.337	11.10%
Gender	Male > female	8	−0.362	−0.57	−0.154	0.16	33.50%
	Male < female	3	−0.296	−0.634	0.042	0.415	0.00%
Frequency (times/week)	1 time/week	8	−0.254	−0.453	−0.056	0.556	0.00%
	2–3 times/week	3	−0.692	−1.084	−0.3	0.255	26.80%
	4–8 times//week	4	−0.344	−0.636	−0.052	0.387	1.00%
Time (min)	60 ≤ min ≤ 90	5	−0.346	−0.587	−0.105	0.054	57.10%
	90 < min ≤ 150	2	−0.329	−0.914	0.256	0.831	0.00%
	150 < min ≤ 180	3	−0.493	−0.929	−0.058	0.695	0.00%
Duration (week)	1–8 weeks	5	−0.31	−0.543	−0.078	0.06	55.70%
	8 weeks	3	−0.322	−0.673	0.028	0.350	4.80%
	8–16 weeks	6	−0.388	−0.603	−0.172	0.07	51.00%
Event	Mindfulness	6	−0.388	−0.603	−0.172	0.07	51.00%
	Yoga	5	−0.253	−0.564	0.057	0.787	0.00%

“*—” Heterogeneity test cannot be conducted due to the lack of literature*.

#### Effect of Mind–Body Exercises on Patients With PTSD and Anxiety

**Figure 6:**
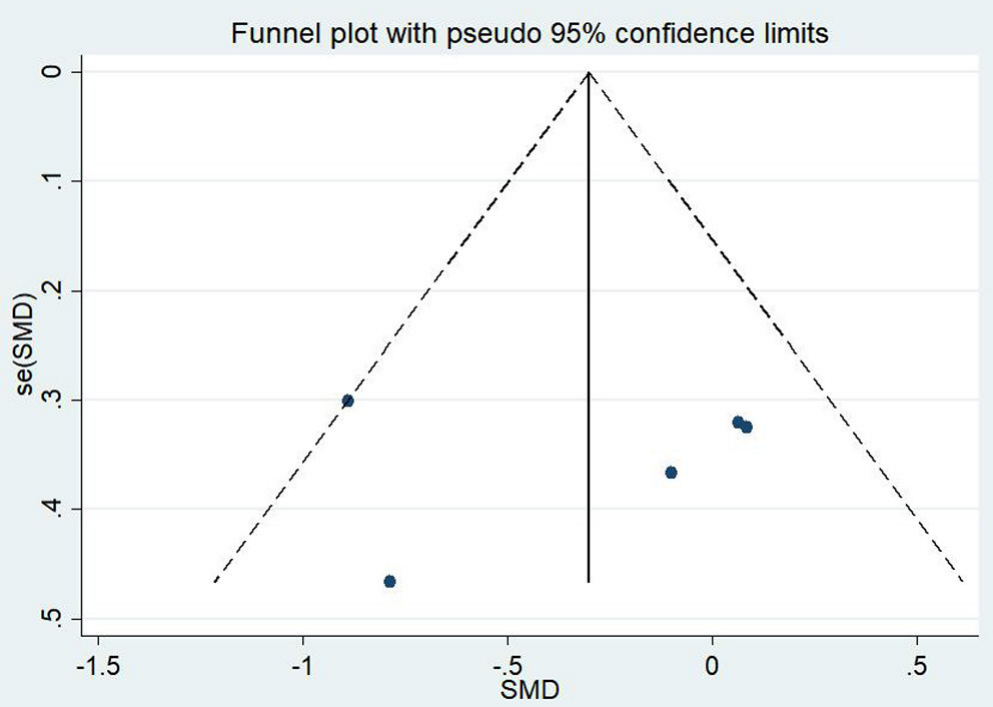
Funnel plot of publication bias for anxiety.

**Figure 7:**
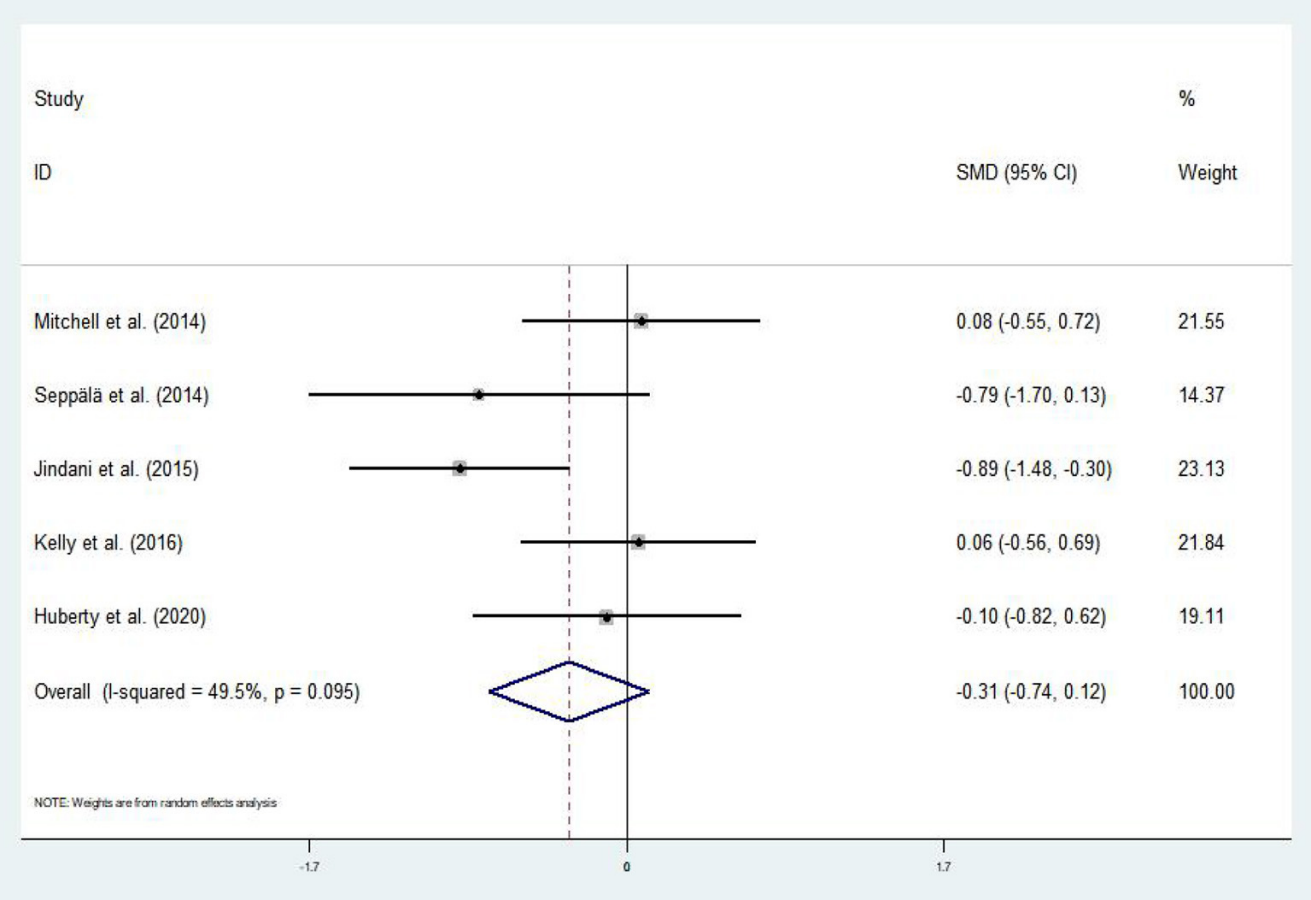
Effect of mind–body exercises on anxiety.

Out of the 16 eligible articles, only five (Mitchell et al., 2014; Seppälä et al., 2014; Jindani et al., 2015; Kelly and Garland, 2016; Huberty et al., 2020) assessed the effect of mind–body exercises on anxiety symptoms in patients with PTSD. The asymmetric funnel plot in Figure 6 indicated no outliers. The results of the heterogeneity test displayed moderate heterogeneity (*p* = 0.095, *I*^2^ = 49.5%); thus, we employed the random-effects model (Figure 7). Meta-analysis revealed that mind–body exercises significantly improved anxiety symptoms in patients with PTSD (SMD = −0.31, 95% CI: −0.74 to −0.12, *p* < 0.001).

## Discussion

This systematic review and meta-analysis aimed to analyze the effects of mind–body exercises on PTSD symptoms, depression, and anxiety in patients with PTSD and to lay the foundation for the development of rational exercise prescriptions. The results suggested that mind–body exercises can improve PTSD symptoms, depression, and anxiety in patients with PTSD. When a patient's physical condition permits, medical staff can use mind–body exercises as an adjunct to conventional treatments to further optimize the treatment plans for patients with PTSD.

The findings of this systematic review are consistent with those of published articles that concluded that mind–body exercises can improve PTSD symptoms, depression, and anxiety in patients with PTSD. Specifically, the effect size of PTSD, depression, and anxiety ranged from −0.64 to −0.19 (*p* < 0.001), from −0.5 to −0.15 (*p* < 0.001), and from −0.74 to −0.12 (*p* < 0.001), respectively. In addition, a high level of heterogeneity may exist in the study of Kim et al. (2013), who recruited only 22 participants and exhibited an attrition rate as high as 14.3%. Thus, the lower sample content of the final experiment may be the major reason for the heterogeneity.

Subgroup analysis indicated that mindfulness exercises for 60–150 min per session over a span of 8–16 weeks resulted in further significant improvements in PTSD symptoms in patients aged <45 years. In the subgroup analysis for frequency, exercises conducted 1–3 times per week were found significant. In addition, mind–body exercises may be more effective in improving PTSD symptoms among males. For depression, once per week, 4–8 times per week, 60–90 min, or 150–180 min of yoga displayed larger effects on patients with PTSD who were aged more than 45 years. Unfortunately, RCTs on the effect of mind–body exercises on anxiety symptoms in patients with PTSD are limited. Therefore, we were unable to determine the most effective frequency, duration, experimental period, and type of intervention. However, the exercise prescription should follow the recommendations provided by the International Organization of Physical Therapists in Mental Health for the generally healthy population. These recommendations include 150 min of moderate-intensity exercises or 75 min of vigorous exercises per week (Vancampfort et al., 2012). Notably, in the subgroup analysis of PTSD and depression, yoga exhibited differences in heterogeneity for the PTSD (*I*^2^ = 54.5%) and depression (*I*^2^ = 0.0%) subgroups. The mechanisms by which PTSD symptoms and depression occur in patients with PTSD may be the main source of the outcome bias.

PTSD symptoms can inflict a serious impact on a patient's normal social and daily life. However, low-to-moderate-intensity mind–body exercise intervention exerts a positive impact on PTSD symptoms (Telles et al., 2007).

Unhealthy lifestyles and personal traumatic experiences are expected to exert adverse effects on the body, which leads to an imbalance in the HPA axis (McEwen, 2002). Dysfunction in the HPA axis, which is characterized by abnormally high levels of corticotropin-releasing hormone (CRH) and low cortisol levels, is one of the unique neuroendocrine characteristics that distinguish PTSD from other mental diseases (Yehuda, 2002; Yehuda and Golier, 2009). Many studies have demonstrated that cortisol plays a crucial role in the pathophysiology of PTSD. However, among healthy individuals exposed to an acute stressor, the HPA axis can respond to the stressor by secreting relevant hormones, such as the CRH from the hypothalamus, adrenocorticotropic hormone (ACTH) from the anterior pituitary, and glucocorticoids from the adrenal cortex (Sapolsky et al., 2000). Increased basal levels of cortisol can improve declarative memory and performance and are associated with the normalization of glucose metabolism in the limbic system (Twamley et al., 2004). Patients with PTSD symptoms exhibit low basic levels of cortisol (Boscarino, 1996). Compared with individuals without PTSD, the sensitivity of the negative feedback inhibition of cortisol in the HPA axis is higher (Yehuda, 2002). In patients with PTSD, elevated basal cortisol levels are associated with improvement in symptoms (Aerni et al., 2004). Several related studies indicated that exercise is related to the transient increase in plasma cortisol (Brandenberger and Follenius, 1975) and improvement in cognitive function (Twamley et al., 2004). Conversely, CRH secretion is abnormally high in patients with PTSD (Bremner et al., 1997), but the level of ACTH is low, which is the hormone that causes the adrenal cortex to secrete glucocorticoids (Smith et al., 1989). The slow response of ACTH to CRH may lead to a decrease in ACTH secretion in the pituitary, which leads to a decrease in the production of cortisol in the adrenal cortex (Smith et al., 1989). Dehydroepiandrosterone sulfate (DHEAS) is an anabolic steroid molecule that enhances an individual's resistance to stress by preventing cell death and behavioral deficits through neuroprotective effects. Relevant studies have illustrated that reasonable exercise can effectively increase DHEAS levels (Johnson, 1997; Rasmusson et al., 2004). Therefore, mind–body exercises can effectively improve PTSD symptoms in patients with PTSD.

The main psychiatric abnormalities in patients with PTSD are typically symptoms of depression and anxiety (Javidi and Yadollahie, 2012). An increasing number of studies demonstrate that proper exercise is beneficial for depression and anxiety symptoms, including those in patients with PTSD (Rebar et al., 2015; Schuch et al., 2016). Evidence exists that impaired adult hippocampal neurogenesis (AHN) is related to various neurological diseases, including PTSD, depression, and Alzheimer's disease (Gao et al., 2017; Barnett et al., 2019). Thus, enhancing AHN can effectively reduce the mood symptoms of the depression model, improve memory resolution, and avoid the recovery of panic experience (Sahay et al., 2011; Hill et al., 2015). In the pathogenesis of PTSD, a dysfunction in the AHN leads to the onset of cognitive impairment and related psychiatric symptoms (Malberg, 2004; Catlow et al., 2013; Besnard and Sahay, 2016). Therefore, improving AHN can effectively alleviate cognitive dysfunction and emotional problems related to patients with PTSD.

The tight regulation of AHN is derived from the interaction between environmental factors and endogenous ecotropic regulators (Toda and Gage, 2018). For example, the PI3K/Akt pathway plays an important role in linking pro-neurogenic gene transcription factors with various neurotrophic and growth factors, such as brain-derived neurotrophic factor and epithelial growth factor signaling (Yin H. et al., 2019; Yin L. et al., 2019). Alternatively, several studies provide evidence that highlight the importance of Akt signaling in regulating AHN. In addition, Akt1 is highly expressed in the adult hippocampus and may influence the synaptic plasticity of the CA1 sub-region (Levenga et al., 2017). Scaffold protein disrupted-in-schizophrenia 1 can regulate AHN via the Akt pathway (Wu et al., 2013). Moreover, exercise-mediated Akt signaling may also play a role in cognitive and emotional regulation and promote synaptic plasticity and synapse formation at the same time (Qian et al., 2015; Levenga et al., 2017). Hippocampal neurogenesis is an important neuroplasticity that provides adaptability under stress and regulates cognitive function and antidepressant behavior (Gao et al., 2017, 2018). Thus, a certain density of immature neurons also contributes to the clearance of fear memory, which, in turn, improves memory resolution (Pereira et al., 2014; Johnston et al., 2016).

Previous studies have demonstrated that as a mature non-drug treatment method, psychosomatic movement is reliant on the activation of Akt by AHN to play a role in improving cognition/anti-depression, in promoting AHN, and in improving neurobehavior. In the medical field, scholars are increasingly interested in studying the physical and psychological effects of mind–body interventions, such as mindfulness and yoga. Mind–body exercises can enhance the ability of patients with PTSD to endure unpleasant emotions, which reduces mental stress (Brown and Gerbarg, 2005; Rocha et al., 2012; West et al., 2014). Yoga focuses on a combination of physical exercise and breathing, where the main exercises include body movement, muscle relaxation, and meditation (Granath et al., 2006). Slow and rhythmic breathing in conjunction with yoga body movements can help to activate the parasympathetic nervous system and stimulate vagus nerve activity (Papp et al., 2013), which can effectively alleviate the symptoms of overexcitement. In this manner, this process is beneficial to patients with PTSD. Other studies depicted that yoga can help restore balance to the HPA axis by lowering the levels of the stress hormone cortisol (Gothe et al., 2016). Streeter et al. (2012) proposed that yoga can reduce a stress-induced unsteady state load using three stress response systems, namely, the HPA axis, the autonomic nervous system, and the GABA-ergic system. Therefore, yoga also may be effective in improving the symptoms of patients with PTSD, including depression and anxiety (Kjellgren et al., 2017). Through mindfulness practice, yoga provides practitioners with a strategy for concentration and for enhancing mind and body awareness (Iverson et al., 2011). Specifically, enhancing mindfulness can increase one's acceptance of one's emotions, improve regulation, and reduce avoidance (Mitchell et al., 2014).

In general, patients with PTSD undergo a lengthy disease duration and a high relapse rate; thus, long-term treatment can place a serious financial burden on individuals and families. Estimates suggest that trauma-related disorders cost over $45 billion USD per year in medical and related costs (Tanielian et al., 2016; von der Warth et al., 2020). Thus, mind–body exercises as a complementary alternative therapy can effectively reduce medical expenditures and reduce the burden on society and families. PTSD symptoms may include, but are not limited to, rapid heartbeat, difficulty in breathing, muscle tightness, hyper-arousal, inability to relax, chronic pain, mood issues, racing thoughts, and substance abuse (Briere and Spinazzola, 2005). Low levels of self-esteem, lack of coping skills, and lack of interpersonal skills constitute a range of symptoms that can impact a patient's quality of life and relationships (Van der Kolk, 2006). In this regard, mind–body exercises can effectively relieve muscle tension and improve breathing problems and other related symptoms through physical activities with breathing exercises, which can improve the quality of life of patients to a certain extent.

In the present review, all articles indicated the significant effect of mind–body exercises on PTSD symptoms among patients with PTSD. Specifically, Reinhardt et al. (2017) presented a sample attrition rate of as high as 52.6% and a sample type of veterans. Therefore, many studies face the challenge of retaining veterans for treatment. Moreover, Carter et al. (2013) and Mitchell et al. (2014) suggested that yoga is feasible as a complementary treatment for veterans with PTSD despite the fact that the results of the intervention for PTSD symptoms among veterans failed to consistently indicate significant changes. In summary, additional RCTs are required to demonstrate the effects of mind–body exercises on PTSD symptoms, depression, and anxiety in patients with PTSD. Therefore, mind–body exercises can be used as an alternative treatment for patients with mild forms of PTSD, as well as an adjunct to recovery and home care for patients with moderate or even severe PTSD.

## Limitations

Although the current literature review tentatively established the benefits of mind–body exercises for patients with PTSD, the limitations of the existing studies should be noted. The reliability of the results may have been influenced by age; gender; duration of the disease; frequency, duration, and intensity of the experimental intervention; and type of exercise. Several qualitative differences were observed for the studies included in the review. (1) Several studies employed small sample sizes with very limited age range and/or race and region (many participants were predominantly white). (2) Only three studies indicated that the sample allocation used hidden allocation, which could be a cause of systematic bias in the treatment effect. (3) Only three studies used assessor blinding in measuring the outcomes. (4) The absence of the descriptions of disease duration and degree of illness in the majority of the literature relatively influences the specific analysis of the effectiveness of mind–body exercises on symptom improvement in patients with PTSD. (5) For anxiety, we were unable to determine the optimal frequency, timing, duration, and type of exercise for mind–body exercises for improving anxiety symptoms in patients with PTSD due to the limited number of eligible RCTs. (6) Lastly, methodological differences in the timing, frequency, duration, and outcome measures of the interventions contributed to the differences in outcomes and led to the difficulties in interpretation. Although these issues limit the generalizability of the studies to different populations, the positive correlation between mind–body exercises and PTSD-related symptoms in these studies provides evidence for the generalizability of the findings.

## Conclusions

Research has proven that mind–body exercises can effectively improve PTSD symptoms, depression, and anxiety in patients with PTSD and can be used as an alternative or adjunctive treatment according to the unique situation of each patient. In cases where patients with PTSD can exercise without adverse effects, then medical staff may use mind–body exercises as a supplemental regimen to conventional treatment. In addition, the conclusion of this research requires further confirmation using additional high-quality RCTs with large samples. Meanwhile, the current review can also provide an exercise prescription for relieving stress and traumatic stress disorders for people experiencing PTSD symptoms due to COVID-19.

## Data Availability Statement

The original contributions presented in the study are included in the article/supplementary material, further inquiries can be directed to the corresponding author/s.

## Author Contributions

LL and LW presented the conceptualization and design of the study protocol. LZ, LW, and LL applied the search strategy and screened the literature based on the selection criteria. LZ and X-zL assessed the risk of bias for the screened literature. All researchers were involved in the collation and analysis of the data and interpreted the results of the data analysis. LZ wrote this manuscript, which was further revised and edited by LW, LL, and X-zL. All authors have read the final revised manuscript and agree to its publication.

## Funding

This work was supported by the Ministry of education of Humanities and Social Science project (17YJA890025); the Fundamental Research Funds for the Central Universities (WUT: 2020VI001).

## Conflict of Interest

The authors declare that the research was conducted in the absence of any commercial or financial relationships that could be construed as a potential conflict of interest.

## Publisher's Note

All claims expressed in this article are solely those of the authors and do not necessarily represent those of their affiliated organizations, or those of the publisher, the editors and the reviewers. Any product that may be evaluated in this article, or claim that may be made by its manufacturer, is not guaranteed or endorsed by the publisher.
